# Varicocele treatment in non-obstructive azoospermia: a systematic review

**DOI:** 10.1080/2090598X.2021.1956838

**Published:** 2021-07-26

**Authors:** Stephanie Jensen, Edmund Y. Ko

**Affiliations:** Department of Urology, Loma Linda University Health, Loma Linda, CA, USA

**Keywords:** Non-obstructive azoospermia, varicocele, varicocelectomy, infertility

## Abstract

**Objective**: To review the available literature and identify factors associated with successful outcomes after varicocele repair (VR) in the setting of non-obstructive azoospermia (NOA). **Methods**: The PubMed and EMBASE databases were searched for relevant articles. Primary outcomes were return of spontaneous spermatogenesis, sperm retrieval rates (SRRs), and unassisted and assisted pregnancy rates. Histopathological subtypes, when available, were used for subgroup analysis. **Results**: A total of 16 articles were finally included. The average sample size was 43 and average duration of follow-up was 10.5 months. The average rate of primary spermatogenesis after VR was 27.3%. The average SRR, across five studies in men with NOA undergoing microscopic testicular sperm extraction status after varicocelectomy, was 48.9% vs 32.1% for the untreated cohort groups, and the average spontaneous pregnancy rate was 5.24%. Histopathology subtype was a significant contributing factor when analysed. **Conclusion**: Varicocele repair should be considered in men with NOA, as it may allow some patients to avoid assisted reproductive technologies and improves success rates when utilised.

## Introduction

Male factor infertility affects up to half of all couples struggling to conceive, and 10–20% of men evaluated for infertility are found to be azoospermic [1,2]. Azoospermia is defined as the complete absence of sperm from the ejaculate and the diagnosis requires examination of the pellet of a centrifuged semen sample on at least two occasions [3]. Non-obstructive azoospermia (NOA) is most often a result of primary testicular dysfunction, although endocrine abnormalities are a factor in some cases. Varicoceles are found in 20% of the general male population, in up to 40% of men with infertility, and specifically 4.3–13.3% of men with NOA [4,5]. The role of varicoceles in NOA has not been fully elucidated. The current best practice statement on the evaluation of azoospermic males from the AUA acknowledges that impaired spermatogenesis associated with varicoceles may be reversible but does not give a clear recommendation for management [3]. The treatment of varicoceles in the setting of infertility has been postulated to allow for the induction of spermatogenesis, improvement in spontaneous pregnancy rates, and increased sperm retrieval rates (SRRs) using assisted reproductive technology (ART). This systematic review was performed with the objective of evaluating the utility of varicocele repair (VR) in the setting of NOA and to identify factors that consistently contribute to successful outcomes.

## Methods

### Search strategy

An electronic search for relevant articles was performed using PubMed and EMBASE databases. There were no restrictions placed on the date of publication, and all literature published up until September 2020 was included. Any articles not published in English were excluded. Search terms included non-obstructive azoospermia, varicocele, varicocelectomy and infertility. The Preferred Reporting Items for Systematic Reviews and Meta-Analysis (PRISMA) protocol was used to report our findings [6].

### Eligibility criteria

Studies evaluating the benefit of treating varicoceles in the management of NOA were included in this review. This included prospective and retrospective cohort studies, as well as randomised controlled trials. Systematic reviews, meta-analyses, and studies including patients with obstructive azoospermia or cryptorchidism were excluded. The diagnosis of NOA was based on history and physical examination, hormone levels, and karyotype analysis, as well as two separate pelleted semen samples. For any studies that included ‘virtual’ azoospermia or oligospermia, only the data for the patients with NOA were included. If the data were not reported separately for patients with NOA in the study, it was excluded. Varicoceles were diagnosed based on physical examination, with and without Valsalva. Studies with sample sizes of <10 were excluded.

The primary author reviewed all abstracts obtained that met the above criteria and obtained the full-text publications. If the paper continued to meet all eligibility criteria, the data were extracted and included in the review. Any discrepancies were reviewed and resolved by the second author.

### Outcome measures

Primary outcomes were return of spontaneous spermatogenesis, SRRs, and unassisted and assisted pregnancy rates. Histopathological subtypes, when available, were used for subgroup analysis. Histopathology subtypes included Sertoli cell only (SCO), early maturation arrest (EMA), late maturation arrest (LMA) and hypospermatogenesis (HS). Only patients with palpable varicoceles were included, and categorised as grade I, II and III based on the system introduced by Dubin and Amelar [7]. SRRs were defined as the number of successful retrievals out of total number of attempts. Pregnancy outcomes were categorised as either spontaneous or assisted, indicating use of ART.

### Risk of bias assessment

None of the reviewed studies were randomised controlled trials.

### Analysis

Data on spermatogenesis, SRRs, pregnancy rates, relapse rates and histopathology were collected and pooled to calculate average values.

## Results

**Figure 1.**
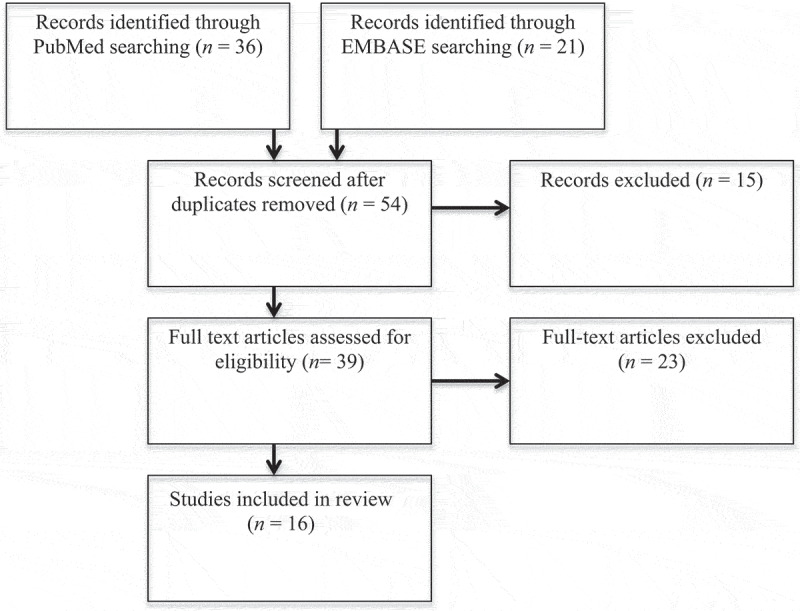
Article selection process

Our electronic search identified 36 papers in the PubMed database and 21 papers in the EMBASE database. Once duplicates were removed, 54 records were screened. After screening the titles and abstracts, 39 of these papers were deemed eligible for inclusion. The full text of these articles was evaluated and only 16 papers adhered to the selected exclusion criteria (Figure 1). Of the papers included in this review, 11 were prospective and five were retrospective. All studies included participants that had a diagnosis of NOA and clinically significant varicoceles, although some studies confirmed varicocele presence with ultrasonography. The average sample size was 43 and mean (range) duration of follow-up was 10.5 (6–22) months (Table 1 [8–23]).

**Table 1.** Summary of data from included articles

Study (first author, year)	Study type	*N*	Varicocele grade, %	Postoperative spermatogenesis, %	SRR, %	Pregnancy rate, %	Relapse rate, %	Histopathology, %	Conclusions
Abdel-Meguid, 2012 [9]	Prospective cohort	31	Grade I – 40 Grade II – 34 Grade III – 26	32.3	–	–	6.5	HS – 41.9 LMA – 19.4 EMA – 6.5 SCO – 32.3	Testicular histopathology was the sole parameter associated with recovery of motile sperm
Aboutaleb, 2014 [18]	Prospective cohort	20	-	30	–	–	–	HS – 35 LMA – 0 EMA – 15 SCO – 50	HS patients have a better chance of SA improvement after VR than MA or SCO patients
Elbardisi, 2019 [12]	Retrospective cohort	42	Grade I – 4.8 Grade II – 47.6 Grade III – 31	26.2	–	–	0	HS – 19.1 LMA – 0 EMA – 21.4 SCO – 59.5	VR in NOA can result in spermatogenesis with highest success expected in HS
Haydardedeoglu, 2009 [21]	Retrospective cohort	96	Grade I – 0 Grade II – 0 Grade III – 100	–	60.8	74.2	–	–	VR should be considered for all NOA with palpable varicocele
Inci, 2009 [22]	Retrospective cohort	96	Grade I – 24 Grade II – 24 Grade III – 35.4	–	53	31.4	–	–	VR in NOA increases SRR in micro-TESE
Kirac, 2012 [17]	Prospective cohort	23	Grade I – 13 Grade II – 39.1 Grade III – 47.9	30.4	–	13	–	–	VR in men with NOA can result in SA improvement, but will likely still require ART
Lee, 2006 [14]**	Prospective cohort	19	Grade I – 10.5 Grade II – 42.1 Grade III – 47.4	36.8	–	5.3	–	HS – 15.8 MA – 31.6 SCO – 52.6	VR should be considered for all NOA patients with palpable varicocele, although benefit in patients with SCO is uncertain
Matthews, 1998 [11]	Prospective cohort	22	Grade I – 12 Grade II – 37 Grade III – 51	55	–	14	–	–	VR resulted in induction or enhancement of spermatogenesis in most men
Ozman, 2018 [20]**	Prospective cohort	32	Grade I – 31.3 Grade II – 59.4 Grade III – 9.3	15.6	–	–	–	HS – 34.4 MA – 31.2 SCO – 34.4	VR can result in improvement of SA, testicular volume was found to be predictive
Pasqualatto, 2006 [19]	Prospective cohort	27	–	33.3	–	3.7	56	HS – 33.3 LMA – 0 EMA – 29.6 SCO – 37	All NOA should be considered for VR, cryopreservation should be discussed due to possibility of relapse
Sajadi, 2018 [16]	Retrospective cohort	57	Grade I – 21.1 Grade II – 42.1 Grade III – 36.8	14	36.8	92	–	–	VR may have positive effect on postoperative spermatogenesis, but effect appears to be more significant on microdissection TESE results
Schlegel, 2003 [15]	Retrospective cohort	31	–	22	–	–	–	HS – 40 MA – 24 SCO – 36	VR will rarely change need for TESE in NOA and should probably be limited to younger men with larger varicoceles
Shiraishi, 2017 [8]	Prospective cohort	83	Grade I – 0 Grade II – 62.7 Grade III – 37.3	24	36	6	–	HS – 16 LMA – 20 EMA – 12 SCO – 52	Cell cycle assessment can predict sperm recovery after VR
Ustuner, 2015 [23]***	Prospective cohort	19	Grade I – 21.1 Grade II – 52.6 Grade III – 26.3	–	–	–	–	HS – 10.5 LMA – 0 EMA – 5.3 SCO – 73.7	VR can result in significant improvement in testicular histology
Youssef, 2009 [13]*	Prospective cohort	54	Grade I – 0 Grade II – 53.2 Grade III – 46.8	34.2	–	3.9	–	Normal – 1.5 HS – 42.6 LMA – 29.6 EMA – 18.5 SCO – 40.7	NOA patients most likely to benefit from varicocele repair are those with HS and LMA
Zampieri, 2013 [10]	Prospective cohort	35	Grade I – 0 Grade II – 0 Grade III – 100	11	57.8	63.2	–	–	VR in NOA significantly increases SRR

SA: semen analysis.

*Varicocele grade data did not differentiate between complete and virtual azoospermia.

**Histopathology categorised as MA without specifying early vs late.

***Subset of patients with histopathology identified as SCO with focal spermatogenesis excluded.

### Presence of sperm in postoperative ejaculate

A total of 13 of the studies assessed spermatogenesis after VR with an average rate of 27.3% [8–20]. For two of those studies, the sperm were specified to be motile [9,11]. The remaining studies included both motile and non-motile sperm. The rate of motility was evaluated in four of the papers, with a mean (range) motility rate of 29 (8–55)% [8–11].

### Sperm retrieval rates

Four of the five studies that evaluated SRRs had a cohort of patients with NOA that had not undergone VR [10,16,21,22]. The average SRR across the five studies for men with NOA undergoing microscopic testicular sperm extraction (micro-TESE) status after varicocelectomy was 48.9% vs 32.1% for the untreated cohort groups [8,10,16,21,22].

### Pregnancy rates

Pregnancy rates were evaluated in nine of the reviewed studies [10,11,13,14,16,17,19,21,22]. The average spontaneous pregnancy rate was 5.24% [11,13,14,17,19]. Of the patients who underwent intracytoplasmic sperm injection (ICSI) after VR, the average pregnancy rate was 65.2%, in comparison to the pregnancy rate for untreated cohort groups following ICSI, which averaged 39.5% [10,16,21,22].

### Azoospermia relapse

Azoospermia relapse rates were examined in three studies with a range of 0% to 56%, with an average rate of 20.8% [9,12,19].

### Histopathology

Testicular histopathology was reviewed in eight of the included papers [8,11–15,18,20,23]. The biopsy samples were obtained either prior to or at the time of varicocelectomy, and specimens were categorised based on histopathological criteria into the following groups: SCO, EMA, LMA, HS and normal spermatogenesis. The only study that found any biopsies consistent with normal spermatogenesis included patients with virtual azoospermia and did not specify how many of the patients with virtual azoospermia fell into each category. Several studies combined EMA and LMA into a general maturation arrest (MA) category. To allow for comparison, the EMA and LMA data, when reported separately, were combined. On average, 49.4% of patients evaluated in these studies were categorised as SCO, 27.6% as MA, and 26.9% as HS. Seven of the eight papers assessed post-varicocelectomy spermatogenesis for each group [9,12–15,18,20,23]. On average, 62.9% of patients with HS were found to have presence of sperm in postoperative semen analysis, as compared to 26.3% of patients with MA and 7.3% of patients with SCO. One study repeated testicular biopsies between 8 and 20 months after varicocelectomy and found that fewer biopsies were consistent with SCO (47.4% from 73.7%) with proportional increases in MA (10.5% from 5.3%) and SCO with focal spermatogenesis (26.3% from 10.5%) categories [23].

### Gene expression

Shiraishi et al. [8] analysed genome-wide mRNA expression levels using transcriptome analysis. Over 23,000 genes were screened, and several of the genes found to be upregulated were noted to be cell cycle related. The level of expression of one of these genes, proliferating cell nuclear antigen (*PCNA*), was evaluated, and the mean number of *PCNA*-positive cells was found to be the only parameter that was significantly associated with sperm recovery. This was in comparison to patient characteristics, laboratory values, and histology results.

## Discussion

Varicocele is one of the most common correctable causes of male infertility. However, infertility associated with NOA and varicocele can be challenging to manage. Even in the absence of karyotype abnormalities and Y chromosome microdeletions, varicocele repair has resulted in variable improvements in spermatogenesis, SRRs, and pregnancy rates [24]. For this reason, there continues to be investigation into factors that may help predict success after varicocelectomy in men with NOA. Interpretation of the data on this topic is difficult as most of the available studies have small sample sizes, are retrospective in nature, and lack a control group. A systematic review of the papers that fit the specified search criteria was performed in order to identify trends in data, as well as areas that warrant further investigation.

Spermatogenesis, SRRs and pregnancy rates after VR were evaluated in most of the included studies. Some of these studies included patients with chromosomal abnormalities. However, when analysed as a subgroup, the data did not differ significantly from the remainder of the cohort [22]. Approximately one-third of patients were found to have sperm present in their postoperative ejaculate, although with a wide range of motility rates. On average, sperm could be retrieved in almost half of the patients after VR in comparison to approximately one-third of the patients in the control groups. This is consistent with the higher pregnancy rates found in those using ART vs control groups. These findings are supportive of the current recommendations to consider treatment of clinically palpable varicoceles when present in patients with NOA. However, there remains a significant portion of patients who fail to benefit from the procedure.

Testicular histology may provide further guidance in determining which patients are most likely to benefit from VR. Patients with histology categorised as HS consistently have higher rates of postoperative spermatogenesis, in comparison with the very low rates seen in the SCO groups. In addition, several authors argue that the patients with SCO with sperm in the postoperative ejaculate were likely incorrectly categorised due to sampling error. As SCO was found in nearly half of the biopsied patients, it is important to discuss the potentially low rate of success when reviewing the risks and benefits of VR. However, Ustuner et al. [23] did note improvement in the histology findings of some patients postoperatively. In patients with a high level of concern or at a high risk of postoperative complications, it may be advisable to obtain a testicular biopsy prior to VR. Also of consideration is the potential for azoospermia relapse, which has been documented, but not frequently investigated.

In regard to future directions, further investigation into the implications of gene expression may help clarify which patients have the best prognosis after VR. However, as varicocelectomy is a very low-risk surgical procedure, it could be argued that the potential benefits of proceeding with VR may outweigh those derived from further evaluations to allow for more accurate preoperative counselling.

## Conclusions

Varicocele repair is the only surgical treatment that has demonstrated the return of sperm to the ejaculate in this patient population, and the results of the present review indicate that it can potentially result in significant improvements in several other fertility parameters including SRRs and pregnancy rates. Although not directly evaluated in every study, the improvement in spermatogenesis rates indicates that varicocelectomy could allow a proportion of patients to avoid the time and cost associated with ART. Additional prospective cohort or randomised controlled trials are needed to further study this specific population.
